# Effect of Duty Cycle on Properties of Al_2_O_3_ Ceramic Coatings Fabricated on TiAl Alloy via Cathodic Plasma Electrolytic Deposition

**DOI:** 10.3390/ma11101962

**Published:** 2018-10-12

**Authors:** Shaoqing Wang, Faqin Xie, Xiangqing Wu, Jixiang An

**Affiliations:** School of Aeronautics, Northwestern Polytechnical University, Xi’an 710072, China; wsq1209@mail.nwpu.edu.cn (S.W.); wxqwsy@nwpu.edu.cn (X.W.); anjixiang@mail.nwpu.edu.cn (J.A.)

**Keywords:** cathodic plasma electrolytic deposition, TiAl alloy, duty cycle, crystallinity

## Abstract

In order to study the effect of duty cycle during the cathodic plasma electrolytic deposition (CPED) process, Al_2_O_3_ ceramic coatings were fabricated via the CPED technique on prepared TiAl alloy in an Al(NO_3_)_3_ electrolyte with different duty cycles. Microstructure, morphology, and chemical compositions of coatings were analyzed by scanning electron microscopy (SEM), energy-dispersive spectroscopy (EDS), transmission electron microscopy (TEM), and X-ray diffraction (XRD). The mechanical properties, such as thickness, hardness, and binding strength, were also characterized, and heat-resistance and wear-resistance tested. The results indicated that duty cycle mainly affected the relative crystallinity of CPED coatings. As the duty cycle increased, the crystallinity of CPED coatings increased, the content of Al(OH)_3_ and γ-Al_2_O_3_ decreased, and the content of α-Al_2_O_3_ increased. The thickness and bonding strength both increased firstly and then decreased, while hardness increased as duty cycle increased. Heat-resistance and wear-resistance of TiAl alloy with CPED coating was highly improved compared to that of TiAl alloy substrate without CPED coating.

## 1. Introduction

Due to the advantages of low density and high specific strength, TiAl alloy could improve the thrust-weight cycle, reliability, life, and safety of the aero-engines significantly. TiAl alloy also has bright prospects with application to aero-engine materials, with lightweight structures and high temperature resistance. However, poor temperature oxidation resistance and wear resistance are important factors restricting applications [1]. Therefore, the surface modification of TiAl alloy is an efficient way to improve heat-resistance and wear-resistance, reduce oxidation, and to maintain excellent performance.

Over the past several decades, researchers have studied the application of thermal spraying, gas deposition, and surface alloying on TiAl alloy to improve performance [2,3,4]. Cathodic plasma electrolytic deposition (CPED), based on plasma micro-arc oxidation, uses the energy of micro-arcs to produce deposits of hydroxide from the surface of the cathode, onto sintered ceramics coatings. The CPED technique overcame the restriction of coating components of substrate metals and fabricated coatings with different compositions in accordance with components of electrolytes [5,6,7,8,9,10]. Xue et al. prepared CPED coatings on the surface of T8 and 304 stainless steels and the results showed that with increasing reaction time, both the wear-resistance and corrosion-resistance of treated steels was improved [11,12]. Liu et al. fabricated alumina ceramic coatings on 304 stainless steel and NiTi alloy and studied the influence of treating frequency of power supply on the microstructure and properties of the coatings [8,13]. However, despite recent progress in the fabrication and performance of CPED coatings, there are few generally accepted theories concerning electrical parameters on the characterization of CPED coating and there is no report studying the effect of duty cycle on the microstructure and properties of CPED coatings.

Herein, the primary objective of this paper is to study the effect of duty cycle on Al_2_O_3_ coatings fabricated on TiAl alloy in Al(NO_3_)_3_ ethanol electrolytes during the CPED process, the influence of duty cycle of power supply on the microstructure and properties of coatings, and the influence mechanism of the duty cycle. This research has been undertaken to enhance our understanding of the CPED technique and to enlarge the scope of its application.

## 2. Experimental Section

### 2.1. Materials

TiAl alloy of nominal composition in a.t.% Ti–47.5Al–1.7V–1.1Cr was used as the substrate and all samples were machined into a size of 20 mm × 10 mm × 5 mm, polished in turn by Grit 160, 240, 400, 600, and 800 SiC abrasive paper, and then cleaned in an ultrasonic bath of acetone.

### 2.2. Fabrication of Al_2_O_3_ CPED Coating

Previous work has evaluated the mechanism and optimized parameters of Al_2_O_3_ CPED coatings on TiAl alloy in Al(NO_3_)_3_ electrolytes [14]. TiAl alloy was preprocessed in electrolytes of Na_2_SiO_3_ (30 g/L), Na_3_PO_4_ (20 g/L), NaOH (3 g/L). A MAO–20C pulse power source was used with pulse frequency and duty cycle fixed at 500 Hz and 20% respectively.

**Figure 1:**
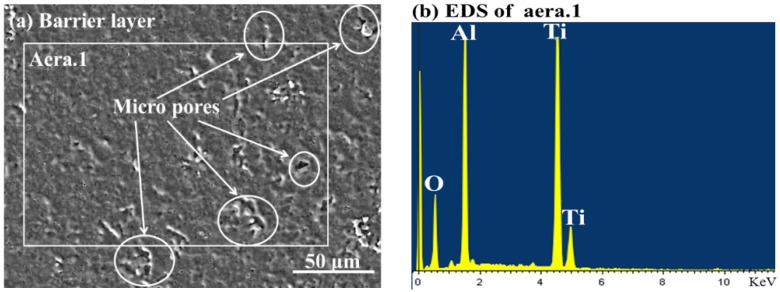
The surface morphology and energy-dispersive spectroscopy (EDS) analysis of the barrier layer.

The surface morphology and energy-dispersive spectroscopy (EDS) analysis of the barrier layer is displayed in Figure 1. It should be noted that there are micro-pores on the uniform and compact barrier layer surface in Figure 1a and the EDS analysis showed that the barrier layer consists largely of elements Al, Ti, and O, which illustrate that the barrier layer was composed of Al_2_O_3_ and TiO_2_.

**Figure 2:**
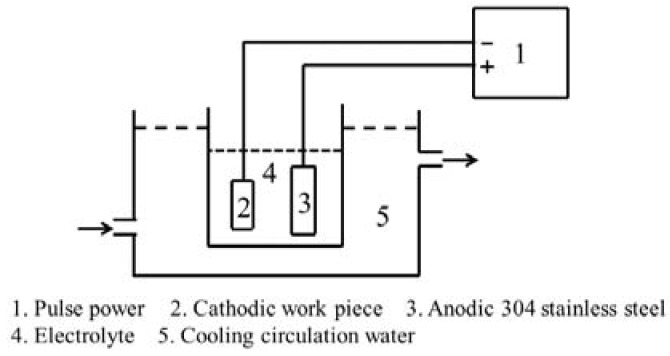
Schematic view of the cathodic plasma electrolytic deposition (CPED) system.

During the CPED process, as shown in Figure 2, a polarity power supply is used. The prepared TiAl samples act as cathodes, with a piece of stainless steel as the anode, in ethanol–water electrolytes with 0.3 M Al(NO_3_)_3_.

The duty cycles are fixed by 5%, 20%, 35%, and 50%, with a stationary current density of 10 A/dm^2^ and a current frequency of 100 Hz. All samples were treated for 30 min. During the CPED process, electrolytes were cooled by circulating water of 3 °C, where the temperature of electrolytes was about 10 °C.

### 2.3. Characterization of Al_2_O_3_ CPED Coating

Scanning electron microscopy (SEM, Verios G4, Philips–FEI Corpocyclen, Netherlands, accelerating voltage: 350 V–30 kV, electron beam: 0.8 pA–100 nA) with energy-dispersive spectroscopy (EDS), transmission electron microscopy (TEM, FEI Talos F200X G2, Philips–FEI Corpocyclen, Netherlands, accelerating voltage: 200 kV, electron gun: Schottky thermal emission), and X-ray diffraction (XRD, XRD–7000, Shimadzu, Japan, XRD, Cu Kα radiation and scanning from 20°–80°) were used to investigate the morphology, high–resolution transmission electron microscopy (HRTEM) micrographs, diffraction patterns, and chemical compositions of Al_2_O_3_ CPED coatings.The hardness and adhesion strength of Al_2_O_3_ CPED coatings were characterized by nano-indenter and profiler (TI–980, Hysitron, America, with 250 g load and dwell time of 30 s) and a bonding strength measuring instrument (WS–2005, Lanzhou Zhongke Kaihua Technology Development Co., China, with dynamic load of 0–100 N).

A HT-1000 pin-on-disk friction and wear tester was used to evaluate wear performance of TiAl alloy and CPED coatings under dry sliding with 10 N load for 15 min, 4 mm wear track radius, and 224 rpm rotating rate against a GCr15 ball, 5 mm in diameter at room temperature. The friction coefficient (μ) was automatically recorded during the sliding wear test. Intermittent isothermal oxidation tests were conducted in static air at 800 °C in alumina crucibles placed in a muffle furnace. The oxidation specimens were taken out of the furnace and cooled to room temperature at various intervals for mass change measurement. The total mass of a specimen, together with the crucible, was recorded.

## 3. Results and Discussion

### 3.1. Effect of Duty Cycle on the CPED Process

**Figure 3:**
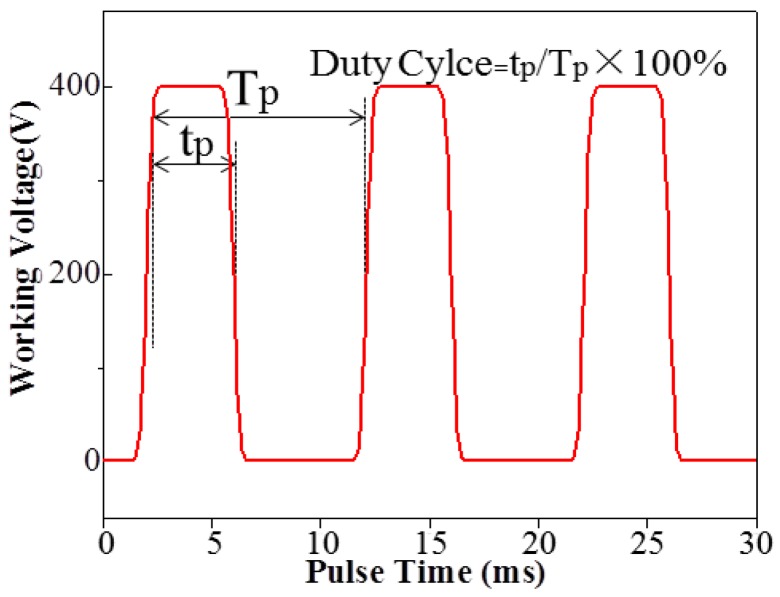
The output oscillogram of impulse voltage.

The output oscillogram of impulse voltage is shown in Figure 3, where duty cycle refers to the percentage of arc discharge time in each impulse cycle. In the paper, the CPED process worked on a 100 Hz frequency with an impulse cycle time of 10 ms. When the duty cycle was fixed at 5%, the arc discharge time was 0.5 ms and the outage time was 9.5 ms. By comparison, when duty cycle was fixed at 50%, the arc discharge time was 5 ms and the outage time was 5 ms.

Each discharge cycle was composed of discharge and outage, due to the special mechanism of the CPED process. Taking Al_2_O_3_ coatings as an example, during the CPED process, Al(OH)_3_ gel particles were adsorbed on the surface of the cathode, sintered, and dehydrated in the period of discharge and then congealed in the period of outage, as to form Al_2_O_3_ ceramic coatings [3,4,5,6].

Al^3+^ + 3OH^−^ → Al(OH)_3_ (1)

2Al(OH)_3_ → Al_2_O_3_ + 3H_2_O (2)

The reactions would be very severe without outages and ceramic coatings would be looser and more porous, or even flawed, due to poor adhesion or being easily cracked or peeled. With the appropriate value of duty cycle, the reaction could proceed smoothly and the CPED coating would be more compact and have a stronger adhesive force.

### 3.2. Characterizations of the CPED Coatings

**Figure 4:**
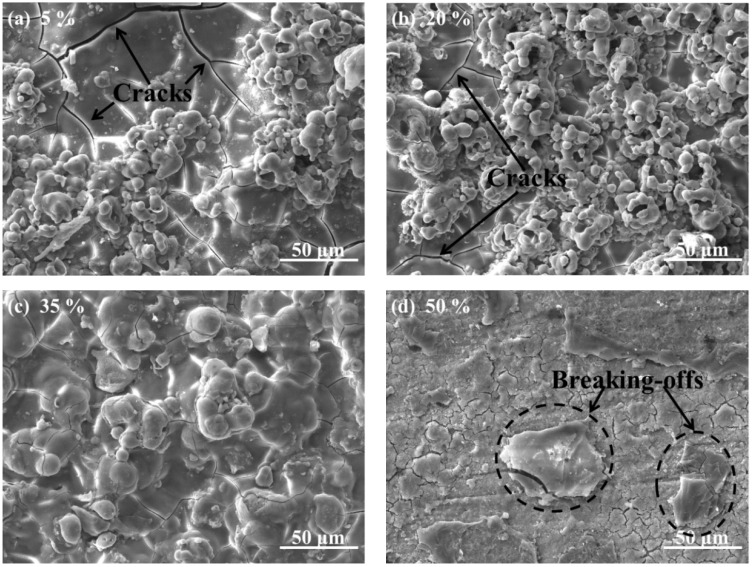
The morphology and the structures of the CPED coatings.

The surface morphologies of CPED coatings under different duty cycles are displayed in Figure 4. When the duty cycle was 5%, the surface of the CPED coating was covered by porosity particles, cracks, and defects (Figure 4a). The uniformity and compactness of the surface improved as duty cycle increased (Figure 4b). When duty cycle was 35%, as shown in Figure 4c with hardly any defects, the coating appeared an uneven or rough surface. However, when duty cycle was increased to 50% there were partial break-offs and the coating had lower uniformity and compactness than that of 20% and 35% (Figure 4d). EDS analysis showed that CPED coatings consisted of the elements Al and O, which illustrate coatings were composed of Al_2_O_3_.

**Figure 5:**
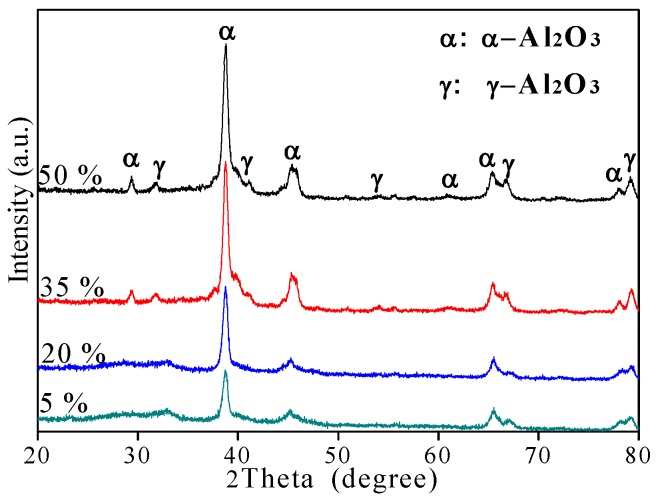
The X-ray diffraction (XRD) patterns of CPED coatings.

The XRD patterns of different CPED coatings are shown in Figure 5 according to the standard chart card (JCPDS PDF No. 10-0425). The phase compositions of the CPED coatings included α-Al_2_O_3_, the main phase, and γ-Al_2_O_3_. When the duty cycle was fixed at 5%, weak diffraction peaks of amorphous structure appeared in a regional scale from 20° to 35° of XRD patterns. It could be inferred that the CPED coating consisted of a small number in the amorphous phase, due to residual Al(OH)_3_ during the CPED process [13,14]. With duty cycle increasing, the intensity of amorphous diffraction peaks became weaker and only sight peaks were detected when the duty cycle was 35%. At the same time, the intensity of α-Al_2_O_3_ diffraction peaks became stronger and the content of α-Al_2_O_3_ increased when duty cycle increased.

**Figure 6:**
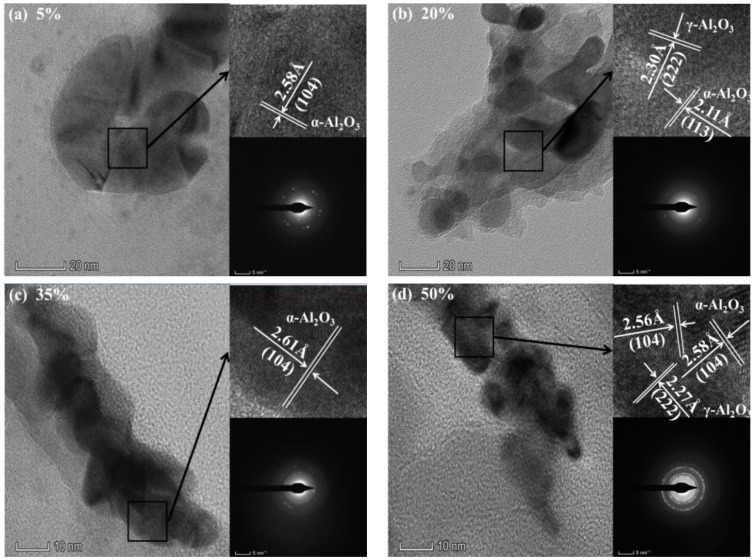
The micro-morphology and electron diffraction patterns of CPED coatings.

Figure 6 shows the micro-morphology and electron diffraction patterns of CPED coatings under high-resolution transmission electron microscopy (HRTEM) under different duty cycles. Figure 6 shows diffraction patterns and rings, amorphous rings, a halo formed by diffuse diffraction, and transmission electron diffraction patterns, indicating that the CPED coatings consisted of nanostructure lattices with a high degree of crystallinity and were well orientated under higher duty cycles. This corresponds to the XRD patterns in Figure 5 and references [9,11,13]. The halo formed by diffuse diffraction was most obvious when the duty cycle was 5%, when scattered diffraction patterns and smaller rings were observed in Figure 6a, and when the crystal lattices displaying CPED coatings included α-Al_2_O_3_ and γ-Al_2_O_3_. It was shown that crystallinity of CPED coatings and the crystal lattice of α-Al_2_O_3_ increased, while that of γ-Al_2_O_3_ decreased, and complete rings were observed in Figure 6b–d with duty cycle rising. When the duty cycle was 50%, as shown in Figure 6c, diffraction patterns of spots and rings were the most obvious and there were different crystal orientations.

### 3.3. The Mechanical Properties and Wear-Resistance of CPED Coatings

**Figure 7:**
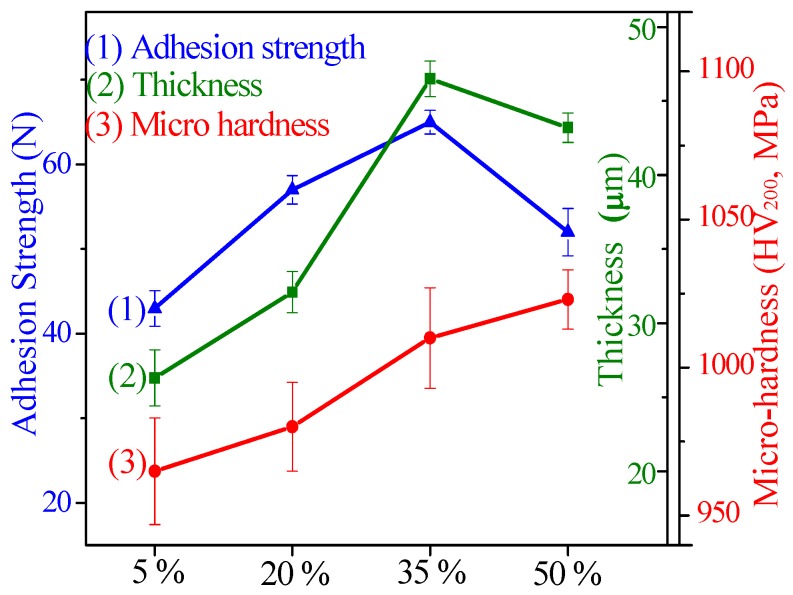
The thickness, micro–hardness, and adhesion strength of CPED coatings.

The thickness, micro-hardness, and adhesion strength of CPED coatings under different duty cycles are shown in Figure 7 and the average value of three parallel samples taken. It can be seen that with duty cycle increasing, the adhesion strength and its relative error both increased firstly and then decreased, and the adhesion strength peaked at 72 N when the duty cycle was 35%. Peaking at 46.5 μm when the duty cycle was 35%, the thickness increased firstly and then decreased, and its relative error continued to fall with duty cycle increasing. When the duty cycle increased to 50%, the micro-hardness showed a growth trend and attained minimal error. When the duty cycle was 5%, the results were identical with the surface morphology of the CPED coatings; there were more defects in the surface, the relative error of thickness was about 7.2% (±1.9 of 26.3 μm), and the relative error of micro–hardness was about 1.87% (±18 of 965 MPa). With duty cycle increasing within a certain scope (under 35%), the surface uniformity of CPED coatings was improved, the thickness, micro-hardness, and adhesion strength increased, and the relative error decreased. When the duty cycle was fixed at 50%, breaking-off occurred, the thickness and adhesion strength declined, and relative error increased, while the value of micro-hardness was bigger and the relative error was smaller.

**Figure 8:**
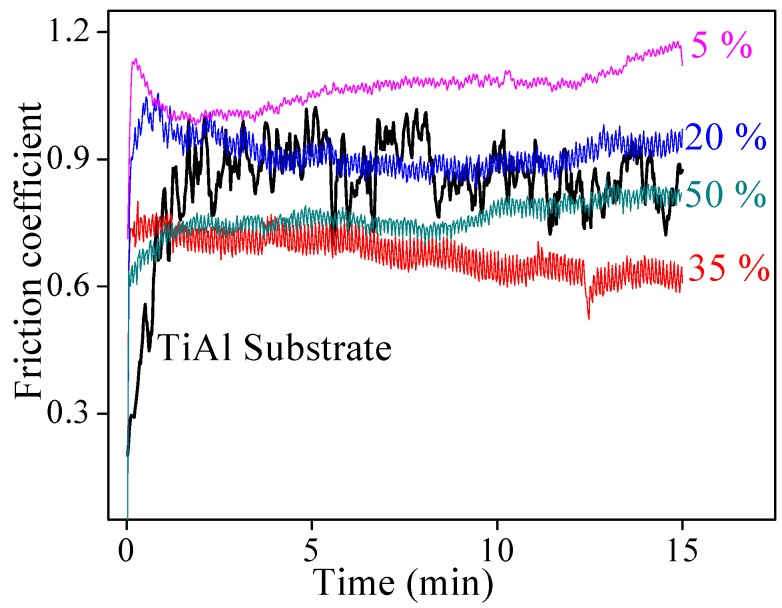
The friction coefficients of TiAl alloy and CPED coatings against GCr15 ball in air under 10 N load for 15 min.

The friction coefficients of TiAl alloy substrate and CPED coatings against the GCr15 ball in air under a 10 N load for 15 min are displayed in Figure 8. During the friction process, adhesive wear occurred for TiAl alloy substrate, where abrasive wear and delamination wear occurred for CPED coatings [15,16]. Compared with TiAl alloy substrate, the running-in periods of CPED coatings were shorter and leveled at more stable friction coefficients. With duty cycle increasing, the friction coefficient decreased substantially and the coating possessed the minimum friction coefficient when the duty cycle was 35%. These results were consistent with analysis of surface morphology of CPED coatings. In the initial stage of the friction process, the clustered bulges in the surface of CPED coatings fractured under cyclic stress and formed debris, this debris acted as solid lubrication between the CPED coating and GCr15 ball and contributed to the shorter running-in periods and more stable friction coefficients. With the duty cycle increasing, the uniformity and compactness of CPED coatings increased and friction coefficients decreased. When the duty cycle was fixed at 50%, the friction coefficient was higher than that at 35% because of higher hardness.

### 3.4. Heat-Resistance of TiAl Alloy Substrate and CPED Coatings

**Figure 9:**
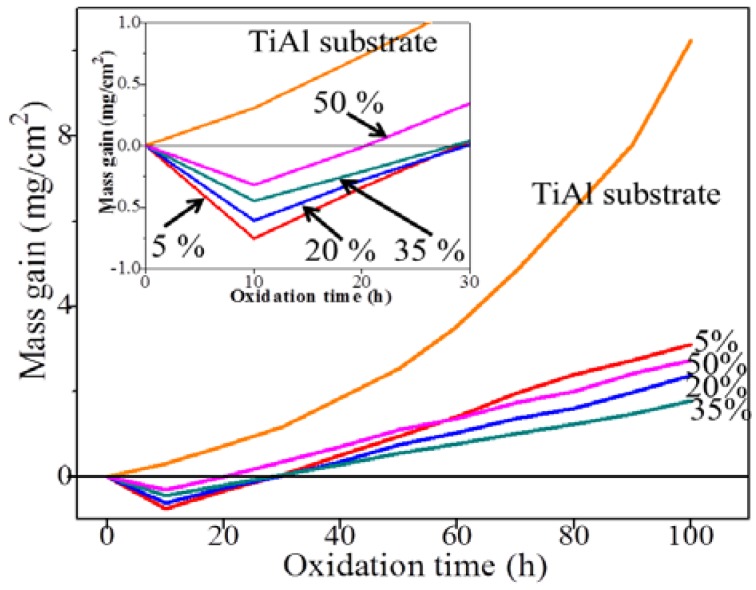
The intermittent isothermal oxidation kinetics curves of TiAl alloy substrate and CPED coatings at 800 °C.

The result of intermittent isothermal oxidation of TiAl alloy substrate and CPED coatings in static air at 800 °C is shown in Figure 9. The oxidation kinetics of TiAl substrate at 800 °C followed a linear law. It was interesting that the mass gain rate of coatings after 10 h oxidation decreased compared to that of the substrate, as shown in Figure 9. The decline was due to the Al(OH)_3_ residues of CPED coating, which was dehydrated under high a temperature [14], with duty cycle increasing, and reduction reduced, illustrating that the content of Al(OH)_3_ decreased and crystallinity of CPED coatings increased. However, when the oxidation time was 100 h, the mass gain rate of CPED coating by 5% was the most and the coating of 35% was the least. This is because when duty cycle was 5%, there were more defects and heat-resistance was the poorest of those coatings. The performance of heat-resistance increased when the uniformity and compactness of the CPED coating increased. When the duty cycle was 50%, breaking-off occurred in the surface of the CPED coating, heat-resistance was weaker than that of 35%, and the mass gain rate was higher.

**Figure 10:**
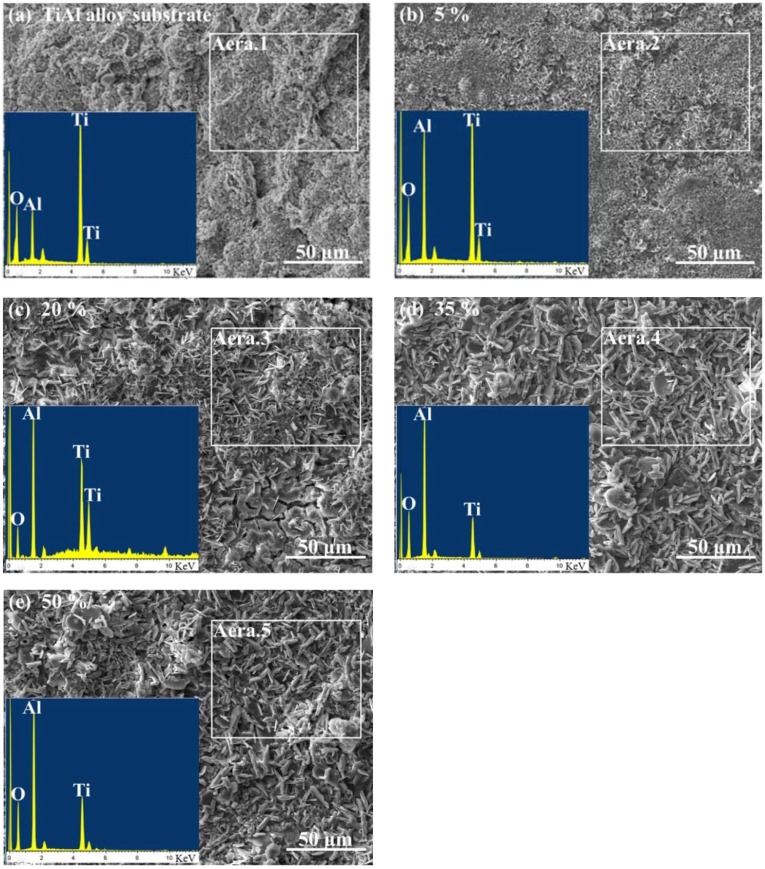
Surface morphology and EDS analysis of TiAl alloy and CPED coatings after an intermittent isothermal oxidation test.

The surface morphology and EDS analysis of TiAl alloy substrate and CPED coatings under different duty cycles after an intermittent isothermal oxidation test are displayed in Figure 10. The porous and loose surface of TiAl alloy after an oxidation test was composed of elements Ti, Al, and O, which meant that the oxidation products of TiAl alloy were made of compounds of primarily TiO_2_ and a little Al_2_O_3_, these results agreed well with previous literature [1,2,3,4]. With duty cycle increasing, the surface of CPED coatings after an oxidation test became denser and more uniform. EDS analysis showed that the amount of Al_2_O_3_ increased, while TiO_2_ decreased. With analysis of surface morphology of CPED coatings in Figure 4 and oxidation kinetics curves in Figure 9, it was discovered that CPED coatings have the advantage of lower oxidation rates than that of TiAl alloy substrate, and that as duty cycle increased so did the performance of heat-resistance.

### 3.5. The Analysis of Mechanism of Duty Cycle

By analyzing properties of CPED coatings as duty cycle increased, the crystallinity and amount of α-Al_2_O_3_ were seen to increase obviously, while cracks and defects in the surface were reduced. Where the uniformity, compactness, and hardness were improved, the adhesion strength and thickness increased at first and then decreased, and the wear-resistance and heat-resistance of CPED coatings were improved compared to TiAl alloy.

The analysis supported that when the duty cycle was small, arc discharge time was short and outage time was long, therefore the action time of electric sparks was also short. There were only a few Al(OH)_3_ gel particles adsorbed on the surface of cathode and a small amount of ceramic particles formed in a single discharge cycle, resulting in more defects in the coating and a smaller thickness and adhesion strength [17,18,19]. Arc discharge time grew longer while outage time became shorter, as duty cycle increased. The Al(OH)_3_ gel particles that adsorbed on the surface sintered and dehydrated adequately in the period of discharge to form Al_2_O_3_ ceramic coatings. The crystallinity of CPED coatings increased, the crystallography orientation became better, and uniformity, compactness, thickness, hardness, and adhesion strength increased by the same [15,16]. The results revealed that the best time proportion between arc discharge and outage properties of the CPED coating were obtained under the duty cycle of 35%. However, under the duty cycle was 50%, there was not enough time for Al(OH)_3_ gel particles to sinter and dehydrate adequately and crystallinity of the CPED coating declined more than under a duty cycle of 35%. This resulted in a decrease in thickness, uniformity, compactness, and adhesion strength.

## 4. Conclusions

(1) Duty cycle mainly affected the relative crystallinity of coatings during the CPED process and the properties of the CPED coating were best under the duty cycle of 35%.

(2) The CPED coatings included α-Al_2_O_3_, γ-Al_2_O_3_ and a little rutile–TiO_2_, and a small number in the amorphous phase Al(OH)_3_. The crystallinity and amount of α-Al_2_O_3_ increased obviously with duty cycle increasing.

(3) With duty cycle increasing, the uniformity, compactness, and hardness of CPED coatings improved, whereas adhesion strength and thickness increased at first and then decreased. The wear-resistance and heat-resistance of CPED coatings was improved compared to TiAl alloy.
